# Editors’ Book Table

**Published:** 1872-09

**Authors:** 


### Editors' Book Table.

[NOTE.—All works reviewed in the columns of the CHICAGO MEDICAL JOURNAL may be found in the extensive stock of W. B. KEEN, COOKE & Co., whose catalogue of Medical Books will be sent to any address upon request.]

BOOKS RECEIVED.

*Hysterology: A Treatise, Descriptive and Clinical, of the Diseases and Displacements of the Uterus.* By EDWIN NESBIT CHAPMAN, M.A., M.D., late Professor of Obstetrics, Diseases of Women and Children and Clinical Midwifery in the Long Island College Hospital. New York: Wm. Wood & Co., 27 Great Jones Street. 1872. Pp. 504.

We have looked over this volume with much interest. It is well illustrated. It elucidates the subject clearly for the student, and the advanced practitioner will be delighted to find in it much valuable information unconnected with womb-burning, slitting and impaling. A book up to the times and for the times.

*The Lettsonian Lectures, Delivered at the Medical Society of London,* 1872, *on the Pathology and Treatment of some Diseases of the Liver.* By S. O. HABERSHON, M.D., Lond., F.R.C.P., Physician to and Lecturer at Guy's Hospital, etc. Philadelphia: Lindsay & Blakiston. 1872. Pp. 91.

Like everything from Dr. Habershon, excellent in principle, clear in diction, satisfactory in practice.

*Thermic Fever, or Sun-Stroke.* By H. C. WOOD, JR., M.D., Professor of Medical Botany, and Clinic Lecturer on Diseases of the Nervous System in the University of Pennsylvania; Physician to the Philadelphia Hospital. Boylston Prize Essay. *Pro bono publico.* Philadelphia: J. B. Lippincott & Co. 1872. Pp. 128.

*The Ten Laws of Health; or, How Disease is Produced and can be Prevented.* By J. R. BLACK, M.D. Philadelphia: J. B. Lippincott & Co. 1872. Pp. 322.

This is intended for a popular treatise on the ground that the profession are bound to inculcate the modes of preserving health, as well as restoring it when impaired. It is well written, and in the main adapted to the purpose in view.

*A Manual of Qualitative Analysis.* By ROBERT GALLOWAY, F.C.S., Professor of Applied Chemistry in the Royal College of Science for Ireland; author of "The Second Step in Chemistry," "The First Step in Chemistry," etc. From the Fifth re-written and enlarged London edition; with illustrations. Philadelphia: Henry C. Lea. 1872. Pp. 402.

A most thorough and complete exposition of the subject, which ought to be in the hands of every medical student, whether before or after entering upon practice.

*The Physiology of Man; Designed to Represent the Existing State of Physiological Science, as applied to the Functions of the Human Body.* By AUSTIN FLINT, JR., M.D., Professor of Physiology and Physiological Anatomy in the Bellevue Hospital Medical College, New York, etc., etc. NERVOUS SYSTEM. New York: D. Appleton & Company, 549 and 551 Broadway. 1872. Pp. 470. [Fourth volume of the Series.]

Notice in preparation.

